# Differentiating malignant and benign necrotic lung lesions using kVp-switching dual-energy spectral computed tomography

**DOI:** 10.1186/s12880-021-00611-6

**Published:** 2021-05-13

**Authors:** Qi Li, Xiao Fan, Tian-You Luo, Fa-Jin Lv, Xing-Tao Huang

**Affiliations:** 1 Department of Radiology, The First Affiliated Hospital of Chongqing Medical University, No. 1 Youyi Road, Yuzhong District, Chongqing, China; 2 Department of Radiology, Children’s Hospital of Chongqing Medical University, No. 136 Zhongshan Road Two, Yuzhong District, Chongqing, China; 3 Department of Radiology, University of Chinese Academy of Sciences Chongqing Renji Hospital (Fifth People’s Hospital of Chongqing), No. 24 Renji Road, Nan’an District, Chongqing, China

**Keywords:** Lung neoplasms, Inflammation, Necrosis, Dual-energy computed tomography, Quantitative imaging

## Abstract

**Background:**

Necrotic pulmonary lesions manifest as relatively low-density internally on contrast-enhanced computed tomography (CT). However, using CT to differentiate malignant and benign necrotic pulmonary lesions is challenging, as these lesions have similar peripheral enhancement. With the introduction of dual-energy spectral CT (DESCT), more quantitative parameters can be obtained and the ability to differentiate material compositions has been highly promoted. This study investigated the use of kVp-switching DESCT in differentiating malignant from benign necrotic lung lesions.

**Methods:**

From October 2016 to February 2019, 40 patients with necrotic lung cancer (NLC) and 31 with necrotic pulmonary mass-like inflammatory lesion (NPMIL) were enrolled and underwent DESCT. The clinical characteristics of patients, CT morphological features, and DESCT quantitative parameters of lesions were compared between the two groups. Binary logistic regression analysis was performed to identify the independent prognostic factors differentiating NPMIL from NLC. Receiver operating characteristic (ROC) curves were used to assess the diagnostic performance of single-parameter and multiparametric analyses.

**Results:**

Significant differences in age, C-reactive protein concentration, the slope of the spectral curve from 40 to 65 keV (K_40–65 keV_) of necrosis in non-contrast-enhanced scanning (NCS), arterial phase (AP) and venous phase (VP), effective atomic number of necrosis in NCS, and iodine concentration (IC) of the solid component in VP were observed between groups (all *p* < 0.05). The aforementioned parameters had area under the ROC curve (AUC) values of 0.747, 0.691, 0.841, 0.641, 0.660, 0.828, and 0.754, respectively, for distinguishing between NLC and NPMIL. Multiparametric analysis showed that age, K_40–65 keV_ of necrosis in NCS, and IC of the solid component in VP were the most effective factors for differentiating NLC from NPMIL, with an AUC of 0.966 and percentage of correct class of 88.7%.

**Conclusions:**

DESCT can differentiate malignant from benign necrotic lung lesions with a relatively high accuracy.

## Background

Necrotic pulmonary lesions manifest as relatively low-density internally on contrast-enhanced computed tomography (CT) [1, 2]. However, the use of conventional CT to differentiate malignant from benign necrotic pulmonary lesions is challenging as these lesions have similar peripheral enhancements. Clinically, the absence of hyperthermia and/or infectious symptoms in necrotic pulmonary mass-like inflammatory lesion (NPMIL) may usually lead to a misdiagnosis. The procedures used to treat NPMIL and necrotic lung cancer (NLC) differ substantially. Necrosis evaluation is inarguably part of necrotic lung mass assessment through which quantitative information of the necrotic area can be obtained. Conventional CT has been widely known to provide valuable morphological information regarding pulmonary lesions [1, 3], but it is unable to supply sufficient quantitative parameters concerning necrosis, except for conventional CT attenuation values, which are not always helpful in determining whether necrotic lung masses are benign or malignant. Therefore, additional tests such as percutaneous lung biopsy and empirical anti-infective therapy are often required.

A novel kVp-switching dual-energy spectral CT (DESCT) imaging mode functioning in a single rotation by completing instantaneous switching between low (80 kVp) and high (140 kVp) voltages to collect data was introduced in 2008 [4]. DESCT can produce virtual monochromatic spectral images at different energy levels, spectral curves, effective atomic numbers, and material decomposition images, thereby improving its ability to differentiate the material compositions [5–12]. Some studies have indicated that benign and malignant necrotic lesions present distinct histological components [13–16]. Therefore, we believe that NPMIL and NLC might be better characterized using DESCT.

To our knowledge, the characteristics of NPMIL and NLC on DESCT images have not yet been established. This study investigates the use of kVp-switching DESCT in the differential diagnosis between benign and malignant necrotic lung lesions.

## Materials and methods

### Patients

**Fig. 1:**
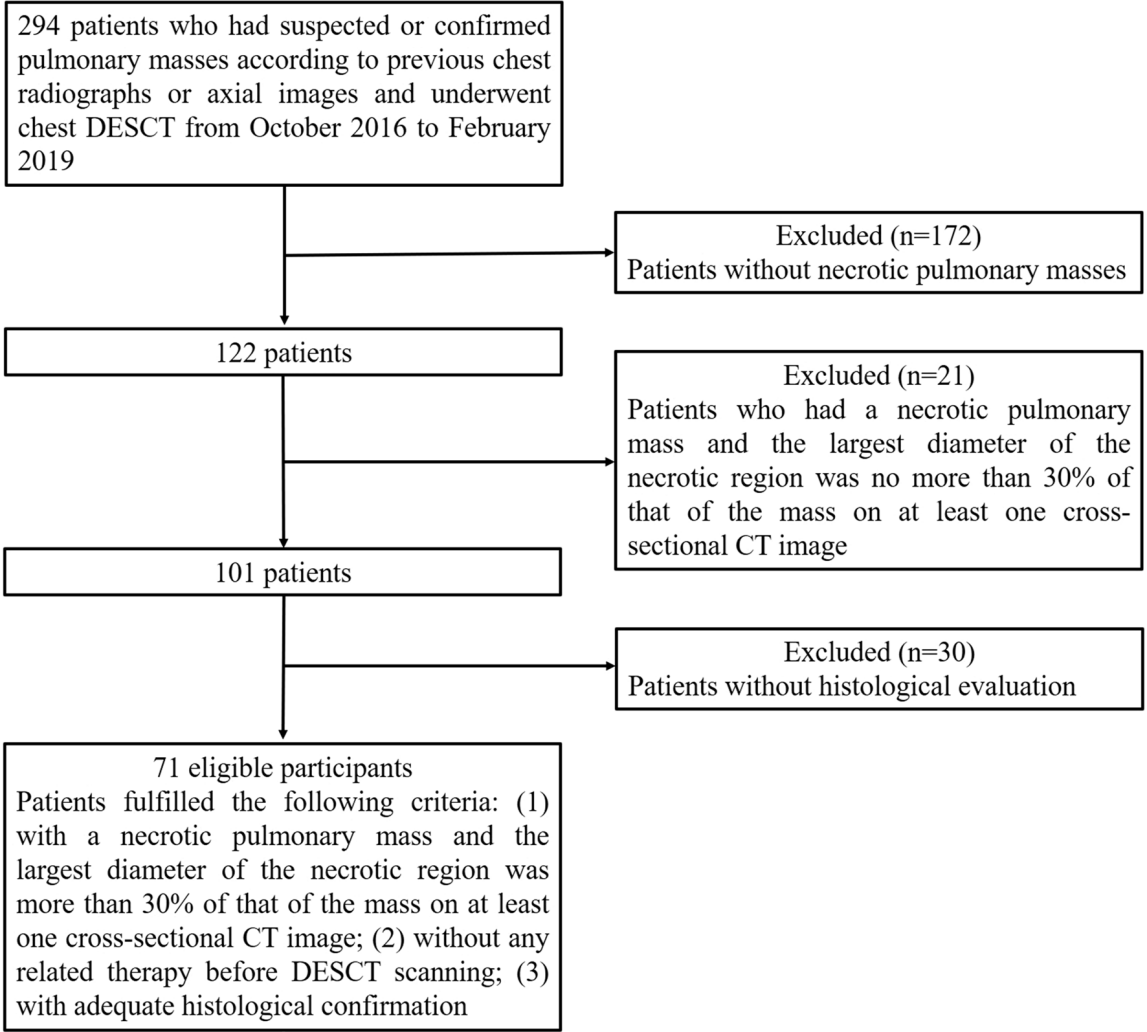
Flow chart of study population

The ethics committee of the First Affiliated Hospital of Chongqing Medical University approved this prospective study, and all patients signed an informed consent for participating in the research at the time of admission. All methods were carried out in accordance with relevant guidelines and regulations. From October 2016 to February 2019, 294 consecutive patients with suspected or confirmed pulmonary masses according to previous chest radiographs or axial images underwent chest DESCT. Subsequently, 223 patients (75.85%) were excluded from the study as 172 patients did not have necrotic lung masses, 21 patients had a necrotic pulmonary mass and the largest diameter of the necrotic region was no more than 30% of that of the mass on at least one cross-sectional CT image, and 30 patients lacked histological evaluation. Finally, the remaining 71 patients (24.15%) participated in this study. These patients fulfilled the following inclusion criteria: (1) patients who had a necrotic pulmonary mass and the largest diameter of the necrotic region was more than 30% of that of the mass on at least one cross-sectional CT image, (2) patients without any related therapy before DESCT scanning, and (3) patients with adequate histological confirmation (obtained through surgical resection, CT-guided percutaneous biopsy, or transbronchoscopic biopsy). The flow chart of study population is shown in Fig. 1. Patients were divided into the following two groups for analysis: Group I consisted of 40 patients with NLC (40 lesions, 12 adenocarcinomas, and 28 squamous cell carcinomas), and Group II consisted of 31 patients (31 lesions, 20 lung abscesses, and 11 tuberculosis). Patients’ clinical characteristics, including age, sex, smoking history, respiratory symptoms, and laboratory results, were recorded.

### CT protocols

All patients were examined using a Discovery CT750HD scanner (GE Healthcare, Milwaukee, WI, USA). Each patient underwent non-contrast-enhanced scanning (NCS) and two-phase contrast-enhanced scanning using the gemstone spectral imaging (GSI) mode. The scanning range of NCS included the whole chest from the first ribs to the diaphragm. Parameters included a detector coverage of 64 × 0.625 mm, tube voltage of 80 kV/140 kV (instantaneous switching), tube current of 260 mA, pitch of 1.375, and CT dose index volume of 10.30 mGy. The patients were then injected with 80–110 mL (1.50 mL/kg of body weight) of nonionic iodinated contrast material (iohexol, 300 mg iodine/mL; Omnipaque, GE Healthcare, Milwaukee, WI, USA) at a rate of 3 mL/s during the arterial phase (AP) and venous phase (VP), which had scan delays of 30 and 60 s, respectively, after contrast medium injection. The scanning range for the AP included the entire chest, whereas that for the VP encompassed only the lesion. The acquisition parameters for the AP and VP were identical to those for NCS [17, 18].

### Imaging analysis

All NCS and two-phase contrast-enhanced scanning images were reconstructed using a slice thickness and interval of 0.625 and 0.625 mm, respectively, for imaging analysis. Two radiologists with > 10 years of experience in chest imaging blinded to patient data interpreted the images and measured the quantitative parameters using the GSI Viewer (GE Healthcare, Milwaukee, WI, USA) together on a workstation (AW4.6; GE Healthcare, Milwaukee, WI, USA). They had several disagreements on six patients when analyzing the CT morphological features of lesions, which were resolved by discussion to reach a consensus. Necrosis was defined as a relatively low-density area within the lung lesions without enhancement on contrast-enhanced CT images [1, 2]. A slice from NCS spectral images for each patient was selected, which matched the same anatomical level of the slices of contrast-enhanced CT images that clearly exhibited necrosis. A circular region of interest (ROI) was drawn to encompass more than half of the necrotic area, avoiding the solid portion. A ROI data file containing the monochromatic CT values at 40–140 keV (with 5 keV increment) and effective atomic numbers was saved for each measurement. To ensure consistency, these measurements were performed at three adjacent image levels, after which the average values of the three measurements from the two reviewers were calculated. The shape and size of the ROIs were made consistent using the copy-and-paste function in each patient. The measuring methods for necrosis in the AP and VP were identical to those for NCS. The placement, shape, and size of the ROIs drawn for three phases were kept as consistently as possible. In addition, the GSI software automatically propagated the ROIs to all 101 sets of monochromatic images with different energies to generate the spectral curves. The slope of the spectral curve between 40 and 65 keV (K_40–65 keV_) for necrosis in three phases was calculated according to the following formula:

$${\text{K}}_{{40{-}65\,{\text{keV}}}} = \left( {{\text{CT}}_{{40{\text{ keV}}}} - {\text{CT}}_{{65{\text{ keV}}}} } \right)/25.$$

Meanwhile, the iodine concentration (IC) of the solid portion in the AP and VP was measured from the iodine-based material decomposition images. Another round ROI as large as possible was placed carefully on the solid region of the lesions in the slice with the most solid components, avoiding necrosis, vessels, or calcification. Similarly, the measurements for IC were performed three times on three consecutive axial images, and the average values were calculated for each patient. Furthermore, the locations, sizes, and CT morphological features of the tumors were observed and recorded.

### Statistical analysis

Statistical analyses were performed using Statistical Package for the Social Sciences, version 19.0 (IBM Corp., Armonk, New York, USA). Single-sample Kolmogorov–Smirnov analysis was performed to test the variance homogeneity of the measurements. Normally distributed quantitative data were expressed as means ± standard deviations, whereas non-normally distributed data were presented as medians ± interquartile ranges. Two-independent-samples Student’s t-test was conducted to assess the parameters consistent with normal distribution; otherwise, the Mann–Whitney U test was used. A two-tailed *P* value < 0.05 was considered statistically significant [17, 18].

Receiver operating characteristic (ROC) analyses of the parameters that differed significantly between groups were performed, which further evaluated their ability to identify NPMIL and NLC by calculating the area under the ROC curve (AUC). Sensitivity, specificity, and Youden Index (YI) (YI_max_ = sensitivity + specificity − 1) were calculated to establish the optimal threshold values. Binary logistic regression was performed using clinical characteristics, CT morphological features, and DESCT quantitative parameters that differed significantly between groups to identify independent prognostic factors of differentiating NLC and NPMIL. The final regression model was selected using the forward condition method, and the AUC was used to evaluate its diagnostic performance.

## Results

### Comparison of clinical characteristics between patients with NLC and those with NPMIL

Fifty-eight men and 13 women were evaluated (age range, 34–84 years; mean age, 58 ± 11 years), of whom 56 (78.87%) were smokers. The clinical characteristics of the patients in both groups are shown in Table 1. The patients with NLC were older than those with NPMIL (*p* < 0.001). Compared to the NLC group, more patients in the NPMIL group had elevated C-reactive protein concentration (*p* < 0.05). However, no significant differences in sex; smoking history; respiratory symptoms, including fever, cough, and chest pain; and white blood cell count were observed between groups (all *p* > 0.05).

**Table 1:** Comparison of patients’ clinical characteristics between groups

Characteristics	NLC (n = 40)	NPMIL (n = 31)	*p* value
Gender			
Men	34 (85.00%)	24 (77.42%)	0.413^a^
Women	6 (15.00%)	7 (22.58%)	
Age (years)			
Mean ± standard deviation	63 ± 9	53 ± 11	< 0.001^b^
Range	43–84	34–79	
Smokers	31 (77.50%)	25 (80.65%)	0.747^a^
Respiratory symptoms			
Fever	4 (10.00%)	6 (19.35%)	0.435^a^
Cough	31 (77.50%)	21(67.74%)	0.357^a^
Chest pain	12 (30.00%)	16 (51.61%)	0.065^a^
Laboratory results			
Elevation of white blood cell count	10 (25.00%)	8 (25.81%)	0.938^a^
Elevation of C-reactive protein concentration	17 (42.50%)	25 (80.65%)	0.001^a^

NLC, necrotic lung cancer; NPMIL, necrotic pulmonary mass-like inflammatory lesion

^a^ Chi-squared test

^b^ Two-independent-samples Student’s t-test

### Comparison of CT morphological features between NLC and NPMIL

The CT morphological features of the lesions in both groups are shown in Table 2. For both groups, the upper lobe was the most common location (63.38%, 45/71), followed by the lower upper lobe (32.39%, 23/71) and the right middle lobe (4.23%, 3/71). However, no significant differences in the locations, lesion and necrotic foci sizes, lesion shapes and margins, and adjacent consolidation or ground-glass opacity were found between groups (all *p* > 0.05).

**Table 2:** Comparison of computed tomography morphological features of lesions between groups

Characteristics	NLC (n = 40)	NPMIL (n = 31)	*p* value
Location*			
The upper lobe	25 (62.50%)	20 (64.52%)	0.861 ^c^
Right upper lobe	11	14	
Left upper lobe	14	6	
The middle and lower lobes	15 (37.50%)	11(35.48%)	
Right middle lobe	2	1	
Right lower lobe	7	8	
Left lower lobe	6	2	
Longest diameter of lesions (cm)			
Mean ± standard deviation	4.94 ± 0.88	4.72 ± 0.80	0.281 ^a^
Range	3.10–6.90	3.20–6.50	
Longest diameter of necrotic foci (cm)			
Median ± interquartile range	2.85 ± 1.10	2.80 ± 1.10	0.280 ^b^
Range	2.10–5.20	2.00–4.30	
Shape			
Round or oval	24 (60.00%)	18 (58.06%)	0.869 ^c^
Irregular	16 (40.00%)	13 (41.94%)	
Margin			
Well-defined	28 (70.00%)	17 (54.84%)	0.188 ^c^
Ill-defined (partially or completely)	12 (30.00%)	14 (45.16%)	
Consolidation or ground-glass opacity around the lesion	17 (42.50%)	18 (58.06%)	0.193 ^c^

NLC, necrotic lung cancer; NPMIL, necrotic pulmonary mass-like inflammatory lesion

^*^If the lesion crosses a fissure, the location is defined as the lobe in which the lesion is predominantly located

^a^ Two-independent-samples Student’s t-test

^b^ Mann–Whitney U test

^c^ Chi-squared test

### Comparison of DESCT quantitative parameters of necrosis in three phases between NLC and NPMIL

**Fig. 2.**
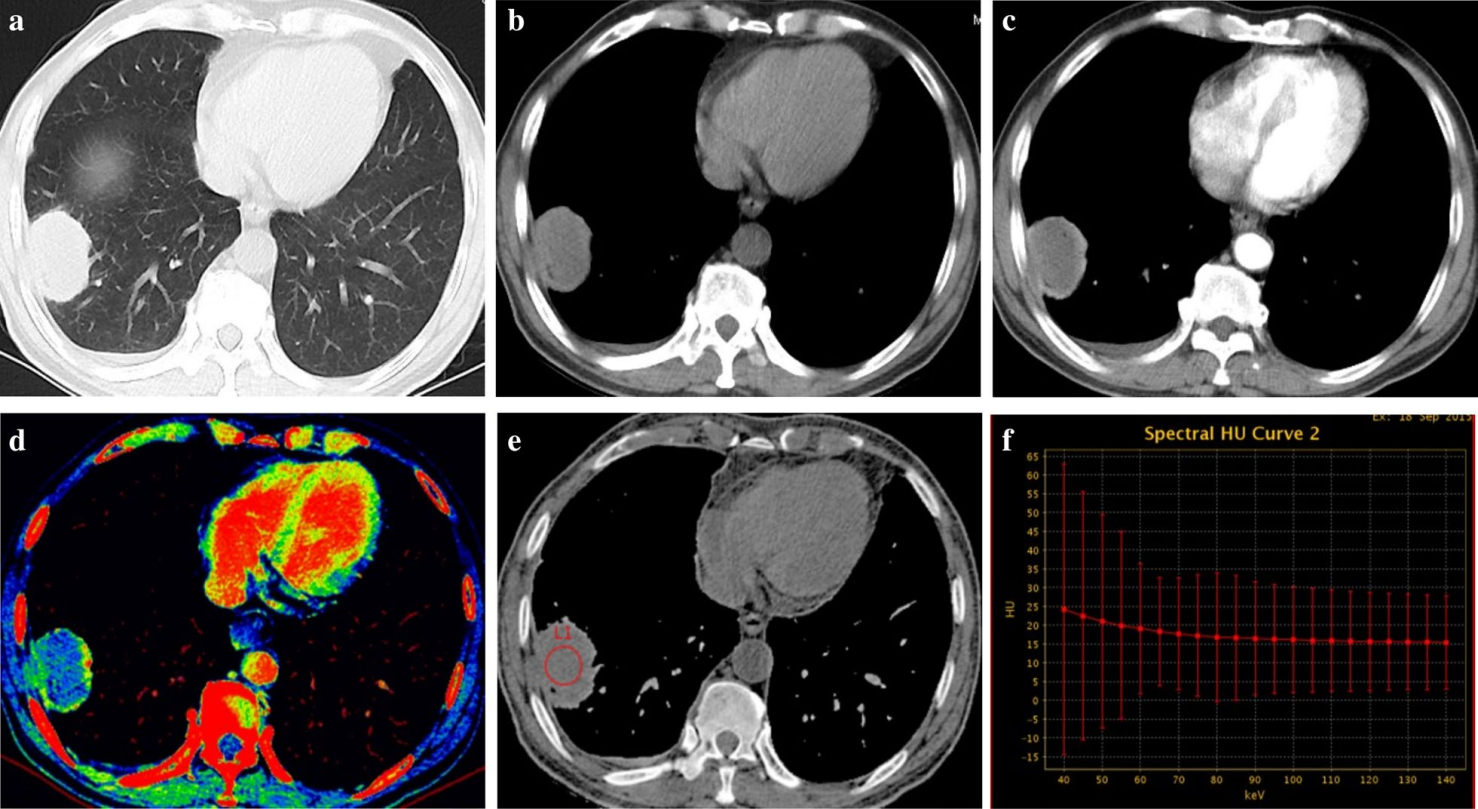
59-year-old male with squamous cell carcinoma. **a** Axial CT image of the lung window showing an oval mass in the right lower lobe. **b**–**d** Axial CT images of the mediastinal window in non-contrast-enhanced scanning (**b**) and arterial phase (**c**), and artificial color map of iodine concentration in venous phase (**d**) showing internal necrosis with ring enhancement. **e** An effective atomic number image showing that the effective atomic number for necrosis in non-contrast-enhanced scanning is 7.63. **f** The gradually declining spectral curve for necrosis in non-contrast-enhanced scanning showing a K_40–65 keV_ of 0.24

**Fig. 3.**
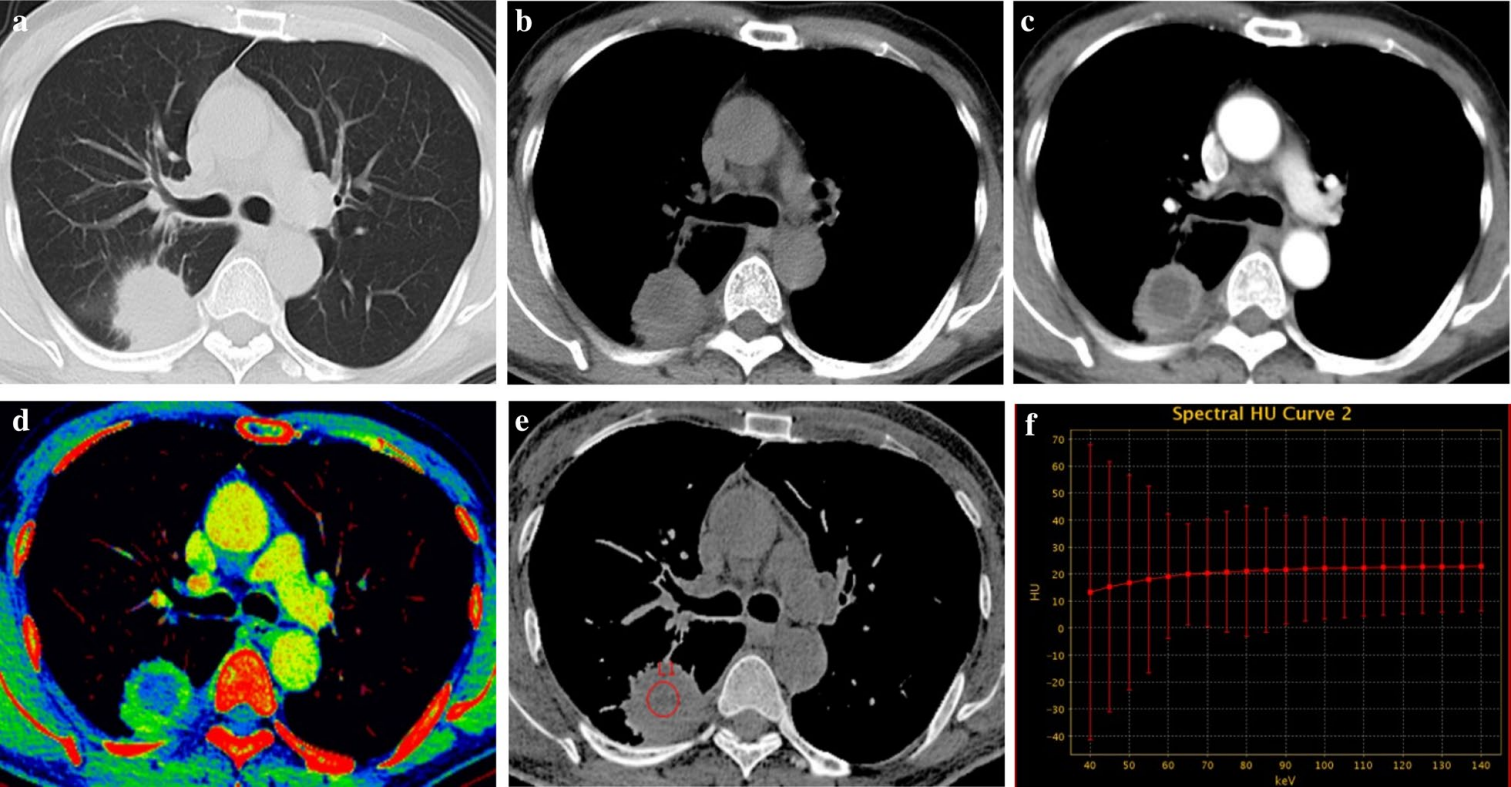
54-year-old male with lung abscess. **a** Axial CT image of the lung window showing a round mass in the right lower lobe. **b**–**d** Axial CT images of the mediastinal window in non-contrast-enhanced scanning (**b**) and arterial phase (**c**), and artificial color map of iodine concentration in venous phase (**d**) showing internal necrosis with ring enhancement. **e** Effective atomic number image showing that the effective atomic number for necrosis in non-contrast-enhanced scanning is 7.44. **f** The gradually increasing spectral curve for necrosis in non-contrast-enhanced scanning showing a K_40–65 keV_ of − 0.43

No significant differences in monochromatic CT values at 70 keV of necrosis in NCS, AP, and VP were observed between groups (all *p* > 0.05) (Table 3). Compared to NPMIL, NLC had significantly higher K_40–65 keV_ in NCS, AP, and VP and effective atomic number in NCS (all *p* < 0.05) (Figs. 2 and 3). However, no significant differences in the effective atomic numbers of necrosis in AP and VP were observed between groups (all *p* > 0.05).

**Table 3:** Comparison of quantitative parameters of necrosis in three phases and iodine concentrations of the solid portion in arterial phase and venous phase with dual-energy spectral computed tomography between groups

Quantitative parameters	NLC (n = 40)	NPMIL (n = 31)	*p* value
**Necrosis**			
NCS			
CT value at 70 keV (HU)	19.16 ± 9.63	15.14 ± 7.66	0.061^a^
K_40–65 keV_	0.31 ± 0.43	− 0.21 ± 0.32	< 0.001^a^
Effective atomic number	7.63 ± 0.14	7.45 ± 0.13	< 0.001^a^
AP			
CT value at 70 keV (HU)	22.80 ± 9.00	19.28 ± 6.95	0.076^a^
K_40–65 keV_	0.29 ± 0.40	0.15 ± 0.34	0.043^b^
Effective atomic number	7.65 ± 0.09	7.63 ± 0.09	0.119^b^
VP			
CT value at 70 keV (HU)	22.29 ± 7.98	19.13 ± 8.54	0.113^a^
K_40–65 keV_	0.34 ± 0.54	0.15 ± 0.38	0.021^b^
Effective atomic number	7.69 ± 0.17	7.68 ± 0.13	0.423^b^
**Solid portion**			
IC in AP (mg/mL)	1.37 ± 0.63	1.41 ± 0.80	0.977 ^b^
IC in VP (mg/mL)	1.48 ± 0.31	1.85 ± 1.01	< 0.001^b^

NLC, necrotic lung cancer; NPMIL, necrotic pulmonary mass-like inflammatory lesion; CT, computed tomography; K_40–65 keV_, the slope of the spectral curve from 40 to 65 keV; NCS, non-contrast-enhanced scanning; AP, arterial phase; VP, venous phase; IC, iodine concentration

^a^ Two-independent-samples Student’s t-test

^b^ Mann–Whitney U test

### Comparison of the IC of the solid portion in AP and VP between NLC and NPMIL

The solid portion of NPMIL had significantly higher IC in VP (*p* < 0.001) than that of NLC (Table 3). No significant difference in the IC of the solid portion in AP was observed between groups (*p* > 0.05).

### ROC and binary logistic regression analyses for differentiating NLC from NPMIL

ROC analyses were performed using the age, C-reactive protein concentration, K_40–65 keV_ of necrosis in NCS, AP, and VP, effective atomic number of necrosis in NCS, and IC of the solid portion in VP in both groups (Table 4). For the model with clinical characteristics, CT morphological features, and DESCT quantitative parameters that differed significantly between groups, the age (odds ratio [OR], 1.203; *p* = 0.001), K_40–65 keV_ of necrosis in NCS (OR, 300.854; *p* = 0.003), and IC of the solid component in VP (OR, 0.002; *p* = 0.002) were proven to be independent prognostic factors that distinguished between malignant and benign necrotic lung lesions via logistic regression analysis. The AUC for this model was 0.966 (95% CI, 0.930–1.000; *p* < 0.001), and the percentage of correct class was 88.7%. With a cut-off value of *p* ≧ 0.746 for diagnosing NLC, the sensitivity and specificity were 0.875 and 1.000, respectively.

**Table 4:** Receiver operating characteristic analyses of clinical characteristics and dual-energy spectral computed tomography quantitative parameters in necrotic lung cancer and pulmonary mass-like inflammatory lesions

Characteristics	AUC (95% CI)	Optimal cut-off value	Sensitivity	Specificity
Age (years)	0.747 (0.626–0.868)	59	0.750	0.742
Elevation of C-reactive protein concentration	0.691 (0.566–0.815)	–	0.806	0.575
Quantitative parameters of necrosis				
K_40–65 keV_ in NCS	0.841 (0.749–0.933)	− 0.12	0.900	0.742
K_40–65 keV_ in AP	0.641 (0.510–0.771)	0.22	0.650	0.645
K_40–65 keV_ in VP	0.660 (0.531–0.790)	0.11	0.875	0.452
Effective atomic number in NCS	0.828 (0.731–0.926)	7.54	0.825	0.774
Quantitative parameters of solid portion				
IC in VP (mg/mL)	0.754 (0.638–0.870)	1.85	0.516	0.925

AUC, area under the receiver operating characteristic curve; CI: confidence interval; K_40–65 keV_, the slope of the spectral curve from 40 to 65 keV; NCS, non-contrast-enhanced scanning; AP, arterial phase; VP, venous phase; IC, iodine concentration

## Discussion

In clinical practice, we have found that many NPMIL may mimic NLC, thus leading to unnecessary surgery in patients with inflammatory masses. Therefore, it is important to differentiate malignant from benign necrotic lung lesions radiographically before treatment. In this study, first, we compared the clinical data and CT morphological features between NPMIL and NLC. Chu et al. [1] found that patients with lung cancers were older than those with inflammatory lesions, which is consistent with our findings. Our results indicated that elevated levels of C-reactive protein were a useful indicator of NPMIL. This can be supported by the evidence that the C-reactive protein is a sensitive and nonspecific inflammatory marker [19]. Furthermore, we found that the CT morphological findings of NLC were similar to those of NPMIL, indicating that conventional CT cannot provide enough information on necrosis, and it is desirable to develop a new imaging technique that can extract additional quantitative information regarding necrosis.

Some investigators have indicated that the CT values on the 70 keV monochromatic images in the GSI mode are similar to those on the 120 kVp conventional polychromatic images [20]. Therefore, we compared the CT values at 70 keV of necrosis in three phases. Our results showed that no significant differences were observed in the CT values at 70 keV in three phases between both groups. Conventional CT generates polychromatic data under a single level of tube voltage. The average attenuation effect of polychromatic X-rays reduces the low-contrast resolution and makes it difficult to separate the materials with small density difference, such as the necrosis of different pulmonary diseases, which may be a good explanation to our findings presented here.

DESCT can supply the spectral curve which represents the changes of monochromatic CT values under different energy levels from 40 to 140 keV. The slope of the spectral curve can be used to evaluate the attenuation characteristics of the X-ray beam passing through different tissues, which are associated with the chemical composition of the constituent substances [7]. Some researchers have used the spectral curve for the differential diagnosis of diseases. A current study [8] indicated a significant difference in the slope of the spectral curve between thymoma and mediastinal lymphoma. Another study [9] showed that the slope of the spectral curve could be used in distinguishing malignant from benign solitary pulmonary nodules. In our study, the slope for necrosis in three phases at low energy levels differed significantly between groups. The possible causes have been attributed to the differences in the histological components of necrotic areas between malignant and benign lung lesions. Lung abscess and tuberculosis have been the most common types of pulmonary benign necrotic lesions, of which necrosis has been associated with inflammatory responses caused by microbial infection [13, 14]. For lung abscesses, necrotic tissues mainly contain highly viscous pus composed of necrotic detritus, inflammatory cells, bacteria, proteinaceous exudate, and fibrinogen [14, 15]. Tuberculosis always uniquely exhibits caseous necrosis characterized by “cheese-like” overall appearance because of its rich lipid content, usually surrounded by lymphocytes and activated macrophages [13]. Unlike inflammatory necrosis, tumoral necrosis usually manifests as coagulative necrosis caused by chronic ischemic injury or hypoxia [13]. Therefore, compared with abscesses, tumoral necrosis is less viscous and contains some tumor cell debris and fewer inflammatory cells [16].

Furthermore, we analyzed the effective atomic number of necrosis, which, in NCS, was found to be significantly higher in the NLC group than in the NPMIL group. If the X-ray attenuation coefficient of the element is the same as that of a compound, the element’s atomic number is the effective atomic number, which can therefore be used to identify the material composition. Indeed, several studies [10, 11] have shown that the effective atomic number plays an important role in differentiating materials, with our results showing the same.

DESCT can provide material decomposition images to quantitatively analyze the composition of a material, which can be manifested as an equal mixture of two basic materials (e.g., iodine and water) after quantitative density measurements [12]. Because iodine is the main component of contrast agents, IC may be a good indicator reflecting the blood supply of pulmonary lesions. Generally, IC and normalized iodine concentration (NIC) are the two most commonly used indicators of iodine concentration in clinic. The IC was derived from the iodine-based material decomposition CT images, while the NIC refers to the ratio of the IC to that in the thoracic aorta [8, 17]. Our previous study showed that IC and NIC in three-phase contrast-enhanced scanning were positively associated with the microvessel density of lung cancer. Moreover, the correlation between IC and microvessel density were better than that between NIC and microvessel density in the same scanning phase. Thus, we used IC to evaluate the blood supply of lesions [17]. This study found that the solid component of the inflammatory lesions showed a higher IC than the cancers in the VP. Previous studies have drawn the same conclusion that the blood supply toward inflammatory lesions is more abundant than that toward tumors [21, 22]. This finding could likely be attributed to the dilation of local vessels resulting from active inflammatory responses or proliferation of inflammatory granulation tissue, whereas malignant tumors usually involve inappropriate angiogenesis and establishment of vascular networks, which may not support the rapid growth of the mass and renders the tumor hypoxic [21, 22].

In this study, we found that the diagnostic performance of the quantitative parameters of necrosis in NCS was much better than that in AP and VP. We speculated that the possible reason for this is that the obvious enhancement of the solid component during contrast-enhanced scanning may change the DESCT quantitative parameters in the adjacent necrotic area because of the partial volume effect. Multiparametric analysis indicated that older age, larger K_40–65 keV_ of necrosis in NCS, and smaller IC of the solid component in VP were the most important independent prognostic factors for diagnosing NLC with an AUC of 0.966. According to our results, the quantitative parameters of the lesions in AP were almost unhelpful in differentiating NLC from NPMIL. Thus, for necrotic lung lesions, we recommend additional NCS or NCS plus contrast-enhanced scanning in VP using the GSI mode, including only the lesion based on conventional scanning sequences.

This study has several limitations worth considering. First, given the small number of patients evaluated here, future studies with larger sample sizes are needed to substantiate our findings. Second, the heterogeneity of the patients in each group was not evaluated because of the small sample size. We are planning to further compare the DESCT characteristics of necrosis between different diseases in each group for a more comprehensive analysis.

## Conclusions

In conclusion, our preliminary results showed that DESCT could provide valuable quantitative information to differentiate malignant from benign necrotic lung lesions. In some cases where diagnosing is difficult, it may be useful for patients to undergo DESCT imaging and gain more quantitative information regarding necrosis, which may contribute to a correct diagnosis and reduction of unnecessary surgery.

## Data Availability

The datasets used and/or analysed during the current study available from the corresponding author on reasonable request.

## Abbreviations

DESCT: Dual-energy spectral computed tomography
CT: Computed tomography
NLC: Necrotic lung cancer
NPMIL: Necrotic pulmonary mass-like inflammatory lesion
GSI: Gemstone spectral imaging
NCS: Non-contrast-enhanced scanning
AP: Arterial phase
VP: Venous phase
ROI: Region of interest
IC: Iodine concentration
K_40–65__ keV_: The slope of the spectral curve from 40 to 65 keV
ROC: Receiver operating characteristic
AUC: Area under the receiver operating characteristic curve
CI: Confidence interval
OR: Odds ratio
NIC: Normalized iodine concentration
